# Association between cumulative social risk, particulate matter environmental pollutant exposure, and cardiovascular disease risk

**DOI:** 10.1186/s12872-020-01329-z

**Published:** 2020-02-11

**Authors:** Ann Canterbury, Justin B. Echouffo-Tcheugui, Daniel Shpilsky, Aryan Aiyer, Steven E. Reis, Sebhat Erqou

**Affiliations:** 1 Department of Medicine, University of Pittsburgh, Pittsburgh, PA USA; 2 Department of Medicine, Johns Hopkins University, Baltimore, MD USA; 3 Department of Medicine, VA Providence Medical Center, Providence, RI USA; 4 Department of Medicine, Alpert Medical School of Brown University, 830 Chalkstone Avenue, Providence, RI USA

**Keywords:** Social risk, Environmental pollutants, Risk factors

## Abstract

**Background:**

Long-term exposure to pollution has been shown to increase risk of cardiovascular disease (CVD) and mortality, and may contribute to the increased risk of CVD among individuals with higher social risk.

**Methods:**

Data from the community-based Heart Strategies Concentrating on Risk Evaluation (HeartSCORE) study were used to quantify Cumulative Social Risk (CSR) by assigning a score of 1 for the presence of each of 4 social risk factors: racial minority, single living, low income, and low educational status. 1-year average air pollution exposure to PM_2.5_ was estimated using land-use regression models. Associations with clinical outcomes were assessed using Cox models, adjusting for traditional CVD risk factors. The primary clinical outcome was combined all-cause mortality and nonfatal CVD events.

**Results:**

Data were available on 1933 participants (mean age 59 years, 66% female, 44% Black). In a median follow up time of 8.3 years, 137 primary clinical outcome events occurred. PM_2.5_ exposure increased with higher CSR score. PM_2.5_ was independently associated with clinical outcome (adjusted hazard ratio [HR]: 1.19 [95% CI: 1.00, 1.41]). Participants with ≥2 CSR factors had an adjusted HR of 2.34 (1.48–3.68) compared to those with CSR = 0. The association was attenuated after accounting for PM_2.5_ (HR: 2.16; [1.34, 3.49]). Mediation analyses indicate that PM_2.5_ explained 13% of the risk of clinical outcome in individuals with CSR score ≥ 2.

**Conclusion:**

In a community-based cohort study, we found that the association of increasing CSR with higher CVD and mortality risks is partially accounted for by exposure to PM_2.5_ environmental pollutants.

## Background

Disparities in health outcomes remain a challenge in the United States [1, 2]. Such disparities are driven by social factors which include ethnicity, income, education, and single living status [3–7]. Multiple epidemiological studies have reported that an accumulation of social risk factors increases the likelihood of cardiovascular disease (CVD) events and deaths [5, 8, 9]. This relationship is thought to be mediated by a myriad of intermediary variables, such as limited healthcare access and utilization, residence in physical and social localities that are detrimental to health, and a combination of health-related behavior and risk factor burden [8, 10–12].

Socially disadvantaged people have increased exposure to unhealthy physical environments [13–15]. Extant evidence indicate that minorities and those who are at increased social risk are more likely to reside in areas close to environmental pollution sources [16, 17]. In addition, studies show that long-term exposure to environmental pollutants, such as ambient fine particulate matter (particles with median aerodynamic diameter < 2.5 μm, [PM_2.5_]), is associated with significantly increased risk for CVD morbidity and mortality and reduced life-expectancy [18–20]. However, the exact contribution of exposure to environmental pollutants to the association between social risk factors and CVD risk largely remains to be clarified.

Accordingly, we sought to evaluate how much long-term exposure to environmental pollutants contributes to increased risk of CVD and mortality in individuals with accumulating social risk factors. We used data from the communit-based Heart Strategies Concentrating on Risk Evaluation (HeartSCORE) study to investigate associations among cumulative social risk (CSR), two common components of urban air pollutants (PM_2.5_ and black carbon [BC]), and the risk of incident CVD and mortality.

## Methods

### Study population

The design of HeartSCORE has been previously described [21]. In brief, HeartSCORE is an ongoing community-based prospective cohort study of racial disparities in CVD that enrolled 2000 participants based in Western Pennsylvania beginning in 2003. The study population is comprised of Black (44%) and White (56%) participants, as well as other minority groups (2.6%). Participants were 45–75 years of age at study entry, had to live in the greater Pittsburgh area, and had to undergo yearly follow-up. Those with co-morbidities that resulted in a life expectancy of less than 5 years were excluded.

### Data collection

Demographic and medical histories were collected at the baseline visit. Participants completed detailed demographic and lifestyle questionnaires including information on self-reported race, marital/co-habiting status, education, income, and smoking. BMI was evaluated by a standard study measurement of weight and height. Blood pressure was measured twice using a manual sphygmomanometer and an appropriately sized cuff after 5 min of rest in a seated position. The average of two readings was taken. Hypertension was defined as a systolic blood pressure ≥ 140 mmHg or a diastolic pressure ≥ 90 mmHg, history of physician-diagnosed hypertension, or current use of anti-hypertensive medication. Lipid panel and glucose were measured in fasting venous blood sample drawn using standard laboratory techniques at the University of Pittsburgh Medical Center clinical laboratory. Diabetes mellitus was defined as fasting glucose ≥126 mg/dL or a history of previously diagnosed diabetes treated with diet, oral agents, and/or insulin.

### Cumulative social risk (CSR)

Cumulative social risk (CSR) was quantified by assigning a score of one for the presence of each of four social factors, as described previously [8, 22, 23] ^9^ i) racial minority ii) single living status, iii) low income, and iv) low educational level. Single living status included those who were not married or cohabiting. Low income was defined as those making <$20,000 a year or those having trouble paying for their basic needs. Participants were classified as low educational level if they did not complete a high school diploma or other equivalent.

### Environmental exposure

Exposure to urban PM_2.5_ and BC was estimated for the year prior to each individual’s baseline assessment, using modified version of land-use regression (LUR) model as previously reported [24–26]. Hybrid LUR models were derived from 37 sampling sites distributed across Greater Pittsburgh area during summer (2012) and winter (2013). Geographic information system (GIS) was used to localize covariates that capture variability in pollution source (e.g., industrial emission, population density) [24]. Hybrid LUR models predicting spatial variation in PM_2.5_ and BC were developed as a function of the GIS-based source density indicators using pollution data from the 37 sampling sites [25]. Particiapnts’ addresses were geocoded using ArcGIS software. The LUR models were then used to estimate the mean concentrations of PM_2.5_ and BC at each participant’s residential address, for the 1-year period preceding date of study entry. Daily regulatory data from a centrally-located U.S. EPA Air Quality System monitor were used to adjust for secular trends in concentration of pollutants [24].

### Clinical outcomes

The primary outcome of interest outcome was a composite of nonfatal CVD events and all-cause mortality. CVD events were defined as nonfatal myocardial infarction, stroke and coronary revascularization (i.e., percutaneous coronary intervention or coronary artery by pass graft). CVD events were monitored by semiannual questionnaires and during annual follow-up study visits. Events were judicated by an independent review of medical records. Mortality was ascertained by reviewing of death certificates.

### Statistical methods

We grouped individuals into the following four categories: CSR = 0, CSR = 1, CSR = 2 and CSR ≥ 3. We assessed the association between CSR and environmental pollutants by plotting the median values of the environmental pollutants across CSR categories. We calculated the *p*-values for trend across CSR categories using the non-parametric Kruskal-Wallis test. We fitted interaction terms between CSR and race in order to evaluate if there was significant effect-modification of the association between CSR and environmental pollutants by race. We used Cox-regression model to determine the association of PM_2.5_ and BC with risk of nonfatal CVD and all cause mortality, with adjustment for established CVD risk factors, namely, age, sex, smoking, systolic blood pressure, diabetes, body mas index, total cholesterol, and HDL-cholesterol. We calculated hazard ratios (HRs) and 95% confidence intervals (95% CIs). We similarly determined the association between CSR and clinical outcome using Cox-regression models. We performed a mediation analyses to assess the potential role of air pollution in explaining the association between CSR and clinical outcomes by adding PM_2.5_ or BC to Cox proportional-hazards models relating CSR and the primary outcome, in a model adjusted for CVD risk factors. The mediation analyses were conducted using the method described by Ananth and VanderWeele, based on the estimated direct and indirect effects calculated for CSR, as computed on the risk difference scale [27]. All analyses were performed using Stata software (Stata Corp., version 11, Texas, USA). *P*-values < 0.05 were considered statistically significant.

## Results

**Fig. 1:**
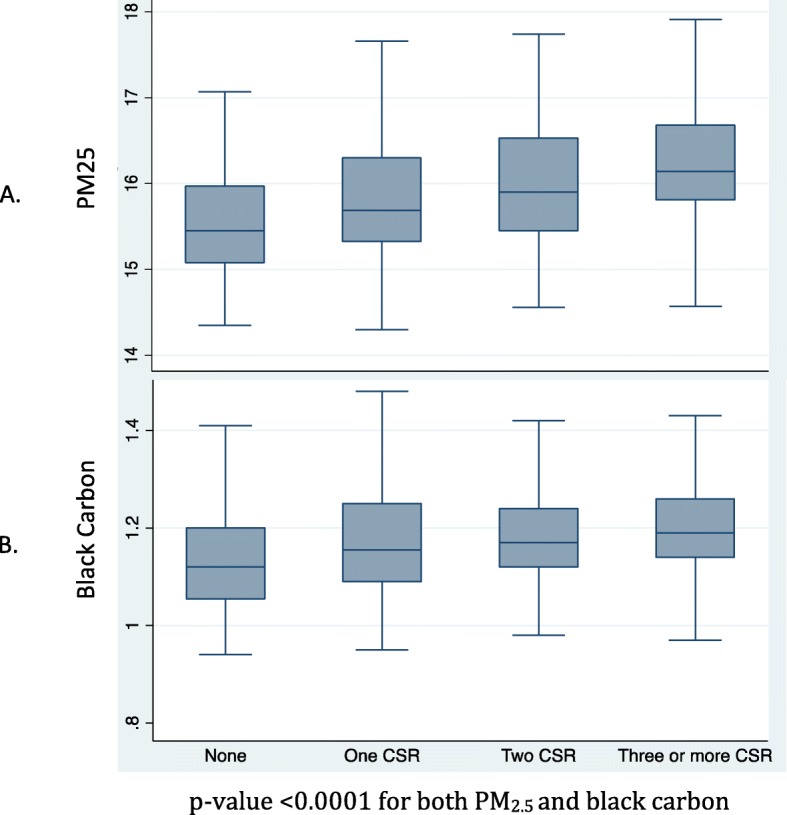
Box plot of PM and black carbon by categories of cumulative social risk. p-value < 0.0001 for both PM_2.5_ and black carbon

Data on CSR were available in up to 1933 participants. The mean (SD) age of participants was 59.0 (7.5) years, with study comprising 66% female and 44% Black individuals (Table 1). In a median follow up time of 8.3 years, (inter-quertile range: 7.1–9.2 years), 137 primary clinical outcome events occurred. Overall, participants with higher CSR score displayed higher levels of traditional CVD risk factors. In addition, increasing number of CSR scores was associated with increased exposure to PM_2.5_. Individuals with CSR score of 0, 1, 2 and ≥ 3 had mean PM_2.5_ concentrations of 15.6, 15.8, 16.0 and 16.3 μg/m^3^, respectively, *P* for trend < 0.001, (Table 1 & Figure). There was a similar pattern of association for BC and CSR (Table 1 & Fig. 1). The associations between CSR and environmental pollutants were not significantly different between Black and White participants (*P* for interaction > 0.05, Additional file 2: Figure S1). Of the components of CSR, ethnicity, income and single living status were similarly associated with PM_2.5_ concentrations, while low education was not statistically associated with PM_2.5_ in univariate models (Additional file 1: Table S1). CSR and PM_2.5_ correlatd with CVD risk factors. Both CSR and PM_2.5_ were similarly associated with higher blood pressure and higher glucose concentrations, although the association between CSR and systolic blood pressure was stronger (Additional file 1: Table S2).

**Table 1:** Baseline characteristics of participants included in study by categories of cumulative social risk*

Variable	N	Overall *N* = 1933	CSR = 0 *N* = 661	CSR = 1 *N* = 565	CSR = 2 *N* = 429	CSR ≥ 3 *N* = 278	p-value
Age (years)	1933	59.0 (7.5)	59.9 (7.3)	59.0 (7.7)	57.8 (7.4)	58.9 (7.4)	< 0.0001
Sex (male)	1933	664 (34.4)	295 (44.7%)	204 (36.1%)	88 (20.5%)	77 (27.7%)	< 0.0001
*Components of cumulative social risk*							
Race (minority)	1933	854 (44.2)	0 (0.0%)	247 (43.7%)	331 (77.0%)	276 (99.3%)	< 0.0001
Income (< low income)	1933	556 (28.8)	0 (0.0%)	98 (17.3%)	183 (42.6%)	275 (98.9%)	< 0.0001
Single living	1933	823 (42.6)	0 (0.0%)	209 (37.0%)	339 (78.8%)	275 (98.9%)	< 0.0001
Education (<high school)	1933	44 (2.3)	0 (0.0%)	11 (1.9%)	7 (1.6%)	26 (9.4%)	< 0.0001
*Environmental pollutants*							
PM_2.5_ (ug/m3)	1661	15.9 (0.8)	15.6 (0.7)	15.8 (0.7)	16.0 (0.7)	16.3 (0.8)	< 0.0001
BC (Abs)	1661	1.2 (0.1)	1.1 (0.1)	1.2 (0.1)	1.2 (0.1)	1.2 (0.1)	< 0.0001
*Cardiovascular risk factors*							
Current smoking	1928	212 (11.0)	43 (6.5%)	45 (8.0%)	64 (14.9%)	60 (21.6%)	< 0.0001
Systolic BP (mmHg)	1931	136.8 (19.7)	133.7 (18.5)	136.0 (18.6)	138.2 (21.1)	143.7 (20.8)	< 0.0001
Diabetes (%)	1923	199 (10.3)	37 (5.6%)	49 (8.7%)	54 (12.6%)	59 (21.3%)	< 0.0001
Hypertension (%)	1930	819 (42.4)	199 (30.2%)	230 (40.9%)	222 (51.6%)	168 (60.4%)	< 0.0001
BMI (kg/m2)	1912	30.2 (6.3)	28.4 (5.2)	30.0 (6.3)	31.1 (6.1)	33.1 (7.8)	< 0.0001
Total Cholesterol (mg/dl)	1921	213.1 (42.8)	214.9 (41.5)	214.1 (41.6)	212.9 (43.6)	207.0 (46.7)	0.076
HDL-cholesterol (mg/dl)	1921	57.6 (15.0)	56.8 (15.5)	57.3 (14.3)	59.1 (15.1)	57.7 (15.1)	0.086

^*^Values are mean (SD) or N (%)

Minority = Blacks in this study; PM_2.5_ = particles with median aerodynamic diameter < 2.5 μm; BC = black carbon, CSR = cumulative social risk

Exposure to PM_2.5_ was associated with an increased risk of all-cause mortality and CVD **(**Table 2a). After adjusting for traditional CVD risk factors (HR: 1.19; 95% CI: 1.00,1.41, *p* = 0.04). BC exposure was not associated with statistically significant increase in risk (HR: 1.12; 95% CI: 0.96,1.31, *p* = 0.14) **(**Table 2b).

**Table 2:** Association of a) PM_2.5_, and b) BC, with combined all-cause mortality and nonfatal CVD (MI, stroke or coronary revascularization) outcomes (*N* = 1620, N cases = 137)

a) PM_2.5_			
Adjustment	HR (95% CI) per 1 SD higher in PM_2.5_	Z-value	P-value
Age & Sex	1.19 (1.01,1.39)	2.1	0.04
Above + smoking	1.17 (1.00,1.37)	1.95	0.05
Above + race	1.14 (0.96,1.34)	1.53	0.13
Above + SBP	1.13 (0.96,1.33)	1.48	0.14
Above + Diabetes	1.15 (0.97,1.35)	1.65	0.1
Above + BMI	1.16 (0.98,1.38)	1.76	0.08
Above + Total cholesterol	1.18 (1.00,1.40)	1.94	0.05
Above + HDL-c	1.19 (1.00,1.41)	2	0.05
Above + TG	1.19 (1.00,1.41)	2.02	0.04
b) Black Carbon			
Adjustment	HR (95% CI) per 1 SD higher in BC	Z-value	P-value
Age & Sex	1.11 (0.95,1.29)	1.35	0.18
Above + smoking	1.11 (0.95,1.29)	1.31	0.19
Above + race	1.09 (0.94,1.27)	1.14	0.25
Above + SBP	1.10 (0.94,1.28)	1.16	0.24
Above + Diabetes	1.09 (0.93,1.27)	1.1	0.27
Above + BMI	1.10 (0.94,1.28)	1.19	0.24
Above + Total cholesterol	1.11 (0.95,1.30)	1.37	0.17
Above + HDL-c	1.12 (0.96,1.30)	1.39	0.16
Above + TG	1.12 (0.96,1.31)	1.46	0.14

Participants with 1, and ≥ 2 CSR scores had age- and sex- adjusted HR of 1.67 (1.06–2.63) and 2.85 (1.84–4.40) for nonfatal CVD event and all-cause mortality, respectively, compared to those with CSR = 0 (Table 3a) The HRs were 1.59 (1.01–2.52) and 2.34 (1.49,3.68), respectively, after adjustment for traditional CVD risk factors in the Full Model. There was similar pattern of association when CSR was fitted as continuous variable (Table 3a).

**Table 3:** Association of cumulative social risk with risk of combined all-cause mortality or CVD outcomes, with adjustment for a) traditional CVD risk factors, b) further adjustment for PM_2.5_ or BC in mediation analyses. (N = 1622, N cases = 137)

a) Traditional CVD risk factors						
	CSR = 1 vs. CSR = 0		CSR ≥ 2 vs. CSR = 0		Test for trend	
Adjustment	HR (95% CI)	Chi-2	HR (95% CI)	Chi-2	HR (95% CI)	Chi-2
Age & sex	1.67 (1.06,2.63)	4.80	2.85(1.84,4.40)	22.28	1.69(1.36,2.10)	22.75
Model 1	1.68 (1.06,2.65)	4.93	2.60(1.67,4.04)	18.06	1.61(1.29,2.00)	18.40
Model 2	1.66(1.05,2.62)	4.67	2.45(1.57,3.82)	15.60	1.56(1.25,1.94)	15.84
Model 3	1.56(0.99,2.48)	3.65	2.20(1.40,3.45)	11.83	1.48(1.18,1.84)	11.97
Model 4	1.57(0.99,2.48)	3.69	2.24(1.42,3.52)	12.04	1.49(1.19,1.86)	12.18
Full Model	1.59(1.01,2.52)	3.96	2.34(1.48,3.68)	13.32	1.52(1.22,1.91)	13.47
b) Mediation analyses						
Full Model + PM2.5	1.54(0.97,2.45)	3.35	2.16(1.34,3.49)	9.92	1.47(1.16,1.86)	10.05
Full Model + BC	1.56(0.99,2.48)	3.61	2.25(1.42,3.58)	11.90	1.50(1.19,1.88)	12.04

Model 1 = Age & Sex + Smoking

Model 2 = Model 1 + SBP

Model 3 = Model 2 + Diabetes

Model 4 = Model 3 + BMI

Full Model = Model 4 + lipid markers (total cholesterol, HDL-c, TG)

The degree of attenuation in the association between CSR and risk of combined all-cause mortality and CVD outcomes when adjusting for PM_2.5_ was comparable to the effect of adjustment for SBP

Mediation analyses indicate that PM_2.5_ explained 13% of the relative risk of CVD and all cause mortality in individuals with CSR score ≥ 2 (compared to those with CSR score = 0). The corresponding value for BC was 7%

Mediation analysis was performed by adding PM_2.5_ or BC to the full model separately (Table 3b). The HR of nonfatal CVD and mortality for participants with CSR ≥ 2 (compared to those with CSR score = 0) were attenuated to 2.16 (1.34,3.49) upon further adjustment for PM_2.5_. This indicates that PM_2.5_ explained 13% of the relative risk of nonfatal CVD and all-cause mortality in individuals with CSR score ≥ 2. The HR was attenuated to smaller degree with adjustment for BC (HR 2.25, 95% CI: 1.42,3.58; Table 3b).

Sensitivity analyses taking CSR in four ordinal categories (i.e., CSR = 0, 1, 2, or ≥ 3) yielded comparable results (Additional file 1: Table S3.). Mediation analyses indicated that PM_2.5_ explained 21% of the relative risk of CVD and all cause mortality in individuals with CSR score ≥ 3 (compared to those with CSR score = 0). The corresponding value for BC was 11%.

## Discussion

In a community-based study of Black and White participants, we found that individuals with higher social risk had higher degree of exposure to environmental pollutants, PM_2.5_ and BC, without evidence of significant effect modification by race. Individuals with increased social risk had substantially higher risk of nonfatal CVD and all-cause mortality. The latter association was partially explained by exposure to PM_2.5._

Multiple epidemiological studies have shown links between social inequality and environment pollution [15, 28, 29]. Furthermore, prior studies have shown that exposure to ambient fine particulate matter increases risk of CVD [4, 18, 20]. In 2010, the American Heart Association released a scientific statement deeming PM_2.5_ a modifiable CVD risk factor [18]. Our study contributes toward a better understanding of the mechanistic pathways of increased CVD and mortality in individuals with higher number of social risk factors. Our findings suggest that exposure to PM_2.5_ may explain approximately 13% of the increased risk in these participants. The mediating effect of PM_2.5_ is comparable to that of blood pressure in our data, which is known to play an important role in CVD risk in the socially disadvantaged [30]. These findings highlight the potential importance of exposure to PM_2.5_ pollutants in mediating adverse outcomes in socially disadvantaged people.

Our findings may have clinical, public health, and policy implications, suggesting that physicians should consider discussions with patients regarding avoidance of environmental pollutant exposure in addition to optimizing other modifiable risk factors such as blood pressure, blood glucose control, and dyslipidemia, especially in socially disadvantaged patients. However, exposure to environmental pollutants is partly related to factors that are usually outside the individual’s control. From a public health and policy perspective, our study highlights the importance of measures that promote cleaner air in neighborhoods as part of the efforts to address social disparities in CVD risk. Further large-scale prospective data is needed, however, to characterize the relative impact of CSR on environmental pollutants and other intermediate CVD risk factors and their relative contribution to CVD and mortality outcomes. Such data can help policy makers in prioritization of resources.

The strengths of the present study merit some consideration. First, this is a community-based study with approximately equal representation of Black and White participants. The study participants were not selected based on preexisting disease, therefore making the findings more applicable to broad populations. Second, our study population was generally associated with a long residence in their current homes, which provided a reliable measure of pollution exposure over time. Third, we were able to use spatial models for air pollution concentrations from a large number of concentration measures collected across the area.

Our investigation has several limitations. First, our sample was drawn from a single geographic area (the Greater Pittsburgh area), which may limit the generalizability of our findings. Second, we did not assess indoor sources of PM_2.5_ and thus may have underestimated the contribution of PM_2.5_ to CVD and mortality risk. However, an important portion of indoor pollution is derived from outdoors [31]. Third, in assessing CSR we dichotomized income and education instead of assessing them in multiple categories; we also assumed that all the components of social risk contribute equally to CSR which may not be necessarily correct. However, this approach has been previously used to simplify complex social exposures and has been shown to be valid in predicting risk of clinical outcomes [8, 22]. Finally, we were not able to evaluate CVD and all-cause mortality separately due to lack of power.

## Conclusion

We found that increased CSR was associated with an increased risk of nonfatal CVD and all-cause mortality. This association was partially accounted for by exposure to fine particulate matter, specifically PM_2.5_. These findings suggest that neighborhood environmental factors are potential targets for intervention at the individual and societal level to reduce adverse health outcomes. Future larger studies are needed to confirm these findings and test that public health effects of relevant interventions.

## Supplementary information

**Additional file 1. Table S1.** Association of social risk and PM_2.5_ with blood pressure levels and blood glucose concentration. **Table S2.** Comparisons of the association of social risk and PM_2.5_ with blood pressure and glucose. **Table S3.** Association of cumulative social risk with risk of combined all-cause mortality or CVD outcomes, with adjustment for a) traditional CVD risk factors, b) further adjustment for PM2.5 or BC in mediation analyses. (*N* = 1622, N cases = 137)

**Additional file 2. Figure S1.** Box plot of PM_2.5_ and black carbon by categories of cumulative social risk, stratified by race

## Data Availability

The datasets used and/or analysed during the current study are available from the corresponding author on reasonable request.

## Abbreviations

BC: Black carbon
CSR: Cumulative social risk
CVD: Cardiovascular disease
GIS: Geographic information system
HeartSCORE: Heart strategies concentrating on risk evaluation
HR: Hazard ratio
LUR: Land-Use regression
PM_2.5_: Particles with median aerodynamic diameter < 2.5 μm
