# Supplementary material for: Association between cumulative social risk, particulate matter environmental pollutant exposure, and cardiovascular disease risk
Source: BMC Cardiovasc Disord. 2020 Feb 11;20:76. doi: 10.1186/s12872-020-01329-z (PMC7014734; doi:10.1186/s12872-020-01329-z)
Supplement: Supplementary file 1 — Additional file 1. Table S1. Association of social risk and PM2.5 with blood pressure levels and blood glucose concentration. Table S2. Comparisons of the association of social risk and PM2.5 with blood pressure and glucose. Table S3. Association of cumulative social risk with risk of combined all-cause mortality or CVD outcomes, with adjustment for a) traditional CVD risk factors, b) further adjustment for PM2.5 or BC in mediation analyses. (N = 1622, N cases = 137) [file 12872_2020_1329_MOESM1_ESM.docx]

**Supplementary Figure 1:** Box plot of PM2.5 and black carbon by categories of cumulative social risk, stratified by race.

*There was no significant effect modification by race
