# Supplementary material for: Association between cumulative social risk, particulate matter environmental pollutant exposure, and cardiovascular disease risk
Source: BMC Cardiovasc Disord. 2020 Feb 11;20:76. doi: 10.1186/s12872-020-01329-z (PMC7014734; doi:10.1186/s12872-020-01329-z)
Supplement: Supplementary file 2 — Additional file 2. Figure S1. Box plot of PM2.5 and black carbon by categories of cumulative social risk, stratified by race [file 12872_2020_1329_MOESM2_ESM.docx]

**Supplementary Table 1**: Association of social risk and PM_2.5_ with blood pressure levels and blood glucose concentration.

| **Variable** | **Social Risk (CSR)** | | **PM_2.5_** | |
| --- | --- | --- | --- | --- |
|  | **Beta (95% CI)** | **P-value** | **Beta (95% CI)** | **P-value** |
| Systolic Blood Pressure (mmHg) | 4.2 (3.1-5.3) | <0.001 | 1.4 (0.2-2.6) | 0.02 |
| Diastolic Blood Pressure (mmHg) | 1.7 (1.1-2.3) | <0.001 | 0.9 (0.2 -1.5) | 0.007 |
| Glucose (mg/dl) | 4.7 (3.1-6.2) | <0.001 | 3.4 (1.7-5.0) | <0.001 |

**Supplementary Table 2**: Comparisons of the association of social risk and PM_2.5_ with blood pressure and glucose.

| **Variable** | **Social Risk (CSR)** | | **PM_2.5_** | |
| --- | --- | --- | --- | --- |
|  | **Beta (95% CI)**  **Per 1 unit** | **P-value** | **Beta (95% CI)**  **Per 1 unit** | **P-value** |
| Systolic Blood Pressure (mmHg) | 4.2 (3.1-5.3) | <0.001 | 1.4 (0.2-2.6) | 0.02 |
| Diastolic Blood Pressure (mmHg) | 1.7 (1.1-2.3) | <0.001 | 0.9 (0.2 -1.5) | 0.007 |
| Glucose (mg/dl) | 4.7 (3.1-6.2) | <0.001 | 3.4 (1.7-5.0) | <0.001 |

**Supplementary Table 3**. Association of cumulative social risk with risk of combined all-cause mortality or CVD outcomes, with adjustment for a) traditional CVD risk factors, b) further adjustment for PM2.5 or BC in mediation analyses. (N = 1622, N cases = 137)

Model 1 = Age & Sex + Smoking

Model 2 = Model 1 + SBP

Model 3 = Model 2 + Diabetes

Model 4 = Model 3 + BMI

Full Model = Model 4 + lipid markers (total cholesterol, HDL-c, TG)

The degree of attenuation in the association between CSR and risk of combined all-cause mortality and CVD outcomes when adjusting for PM_2.5_ was comparable to the effect of adjustment for SBP.

Mediation analyses indicate that PM_2.5_ explained 21% of the relative risk of CVD and all cause mortality in individuals with CSR score ≥3 (compared to those with CSR score = 0). The corresponding value for BC was 11%.

| ***a) Traditional risk factors*** | | | | | | |
| --- | --- | --- | --- | --- | --- | --- |
|  | CSR=1 vs. CSR= 0 | | CSR=2 vs. CSR= 0 | | CSR≥3 vs. CSR= 0 | |
| Adjustment | HR (95% CI) | Chi-2 | HR (95% CI) | Chi-2 | HR (95% CI) | Chi-2 |
| Age & Sex | 1.67(1.06,2.63) | 4.84 | 3.00(1.86,4.85) | 4.50 | 2.63(1.54,4.52) | 12.39 |
| Model 1 | 1.68(1.06,2.66) | 4.97 | 2.78(1.72,4.51) | 4.16 | 2.35(1.36,4.05) | 9.42 |
| Model 2 | 1.66(1.05,2.62) | 4.71 | 2.65(1.63,4.30) | 3.93 | 2.18(1.26,3.78) | 7.67 |
| Model 3 | 1.56(0.99,2.47) | 3.61 | 2.45(1.51,3.98) | 3.61 | 1.86(1.06,3.26) | 4.71 |
| Model 4 | 1.57(0.99,2.48) | 3.69 | 2.47(1.52,4.02) | 3.63 | 1.89(1.07,3.34) | 4.84 |
| Full Model | 1.59(1.01,2.52) | 3.92 | 2.56(1.57,4.18) | 3.77 | 2.00(1.13,3.53) | 5.66 |
|  | | | | | | |
| ***b) Mediation analyses*** | | | | | | |
| Full Model + PM2.5 | 1.53(0.96,2.43) | 3.20 | 2.39(1.44,3.95) | 3.39 | 1.77(0.97,3.23) | 3.42 |
| Full Model + BC | 1.56(0.98,2.47) | 3.53 | 2.49(1.52,4.08) | 3.64 | 1.88(1.06,3.37) | 4.58 |
